# Comprehensive safety analysis of the clinical spectrum of adverse events associated with immune checkpoint inhibitors based on FAERS

**DOI:** 10.3389/fimmu.2026.1823743

**Published:** 2026-05-01

**Authors:** Caihong Qu, Xiaoying Wang

**Affiliations:** 1 Office of Pharmacovigilance, Third Affiliated Hospital of Sun Yat-sen University, Guangzhou, Guangdong, China; 2 Information Center, Third Affiliated Hospital of Sun Yat-sen University, Guangzhou, Guangdong, China

**Keywords:** clinical spectrum of toxicity, disproportional analysis, immune checkpoint inhibitors, immune-related adverse events, pharmacovigilance

## Abstract

**Background:**

The toxicities associated with immune checkpoint inhibitors (ICIs) can affect nearly all organ systems. Although previous studies have examined specific types of immune-related adverse events (irAEs) associated with ICIs, comprehensive evaluations of the clinical profiles of irAEs using data from the FDA Adverse Event Reporting System database over an extended period have been lacking.

**Methods:**

Duplicate records and reports submitted by nonprofessionals were removed for data cleaning. Employing three disproportional statistical methods (Reporting Odds Ratio, Bayesian Confidence Propagation Neural Network, and Multi-Item Gamma Poisson Shrinker) with MedDRA version 27.1 classification, we identified and ranked adverse event signals across System Organ Class (SOC), Standardized MedDRA Query (SMQ), and Preferred Term (PT).

**Results:**

A total of 320,556 adverse event reports associated with eight ICIs, involving 123,210 patients, were included in the analysis. At the SOC level, endocrine disorders and hepatobiliary disorders exhibited the strongest signals, while cardiac and respiratory toxicities showed the highest mortality risks across vital organ systems. At the SMQ level, the most prominent signals included noninfectious encephalitis, hypothyroidism, noninfectious myocarditis/pericarditis, eosinophilic pneumonia, and interstitial lung disease. At the PT level, the leading signals were immune-mediated lung disease, immune-mediated enterocolitis, immune-mediated hepatitis, (immune-mediated) myocarditis, hypophysitis, and adrenal insufficiency.

**Conclusions:**

This pharmacovigilance study provides a systematic analysis of the clinical spectrum and reporting characteristics of ICI-related irAEs, comparing relative risks across vital organ systems and laying the foundation for interdisciplinary collaboration among oncologists and relevant specialists.

## 1 Introduction

Immune checkpoint inhibitors (ICIs) have been approved for the treatment of over 20 tumor types. These include programmed death-1 inhibitors (anti-PD-1), such as nivolumab, pembrolizumab, and cemiplimab; programmed death-ligand 1 inhibitors (anti-PD-L1), such as atezolizumab, avelumab, and durvalumab; and cytotoxic T-lymphocyte-associated antigen-4 inhibitors (anti-CTLA-4), including ipilimumab and tremelimumab. ICIs are indicated for cancers such as melanoma, lymphomas, and malignancies of the kidney, liver, breast, lung, head and neck, urothelial tract, and cervix, particularly in metastatic settings. Since the 2011 approval of ipilimumab, the first ICI targeting CTLA-4, ICIs—often combined with surgery, radiation, chemotherapy, or targeted therapies—have become a cornerstone of cancer treatment. They are widely used to activate the immune response of the body against cancer cells (1).

The administration of ICIs can lead to excessive immune system activation, disrupting immune homeostasis in patients. This dysregulation may result in immune-related adverse events (irAEs), which can affect nearly any organ system. The severity of irAEs associated with various ICIs ranges from mild to severe, with some cases being life-threatening or fatal. Outcomes may be reversible or permanent, and the affected organs and tissues can span from barrier sites to sterile internal environments. As the use of ICIs in oncology expands, clinicians increasingly encounter a broad spectrum of irAEs. A 2019 review categorized irAEs affecting multiple organ systems—including cutaneous, lower gastrointestinal, pulmonary, thyroid, hepatic, cardiac, neurological, ocular, rheumatological, renal, and hematological events, as well as hypophysitis—detailing their epidemiology, management, and kinetics (2). Furthermore, the 2022 European Society for Medical Oncology Clinical Practice Guideline provides evidence-based recommendations for the diagnosis, management, and treatment of specific irAEs, drawing on both scientific literature and clinical experience (3). Nevertheless, managing irAEs of varying severity remains challenging for healthcare providers due to their unpredictable presentation and the diversity of affected organs.

The FDA Adverse Events Reporting System (FAERS) is a publicly accessible database that provides a critical resource for the post-market safety surveillance of pharmaceuticals. It allows for the timely aggregation of rare and serious adverse events (AEs) that may be missed in traditional clinical trials. The extensive volume of data from multiple countries enables the assessment of global trends and the characterization of specific toxicities using robust statistical methodologies. Several studies have explored particular irAEs associated with ICIs using FAERS, including endocrine toxicities (4), psychiatric disorders (5), hepatitis and cholestasis (6, 7), enterocolitis (8), cardiac disorders (9, 10), and interstitial lung disease (11). However, there appears to be a lack of systematic studies examining irAEs across all organ systems over an extended period. To address this gap, we conducted a comprehensive pharmacovigilance analysis of ICI-related irAEs covering all organ systems using FAERS data from 2011 to 2024. This study differentiates itself from previous analyses by providing a comprehensive assessment of these irAEs. Following established formulas and criteria reported in the literature (12, 13), this study examines patient demographic characteristics, the overall distribution of the disease spectrum, and risk comparisons of ICI-related irAEs at multiple levels, including System Organ Classification (SOC), Standardized MedDRA Query (SMQ), and Preferred Term (PT), based on MedDRA version 27.1. The aim of this study was to enhance clinicians’ understanding of ICI-associated toxicities, support multidisciplinary collaborative diagnosis and management, and promote the safe clinical use of ICIs.

## 2 Materials and methods

### 2.1 Data extraction and classification of AEs

**Figure 1:**
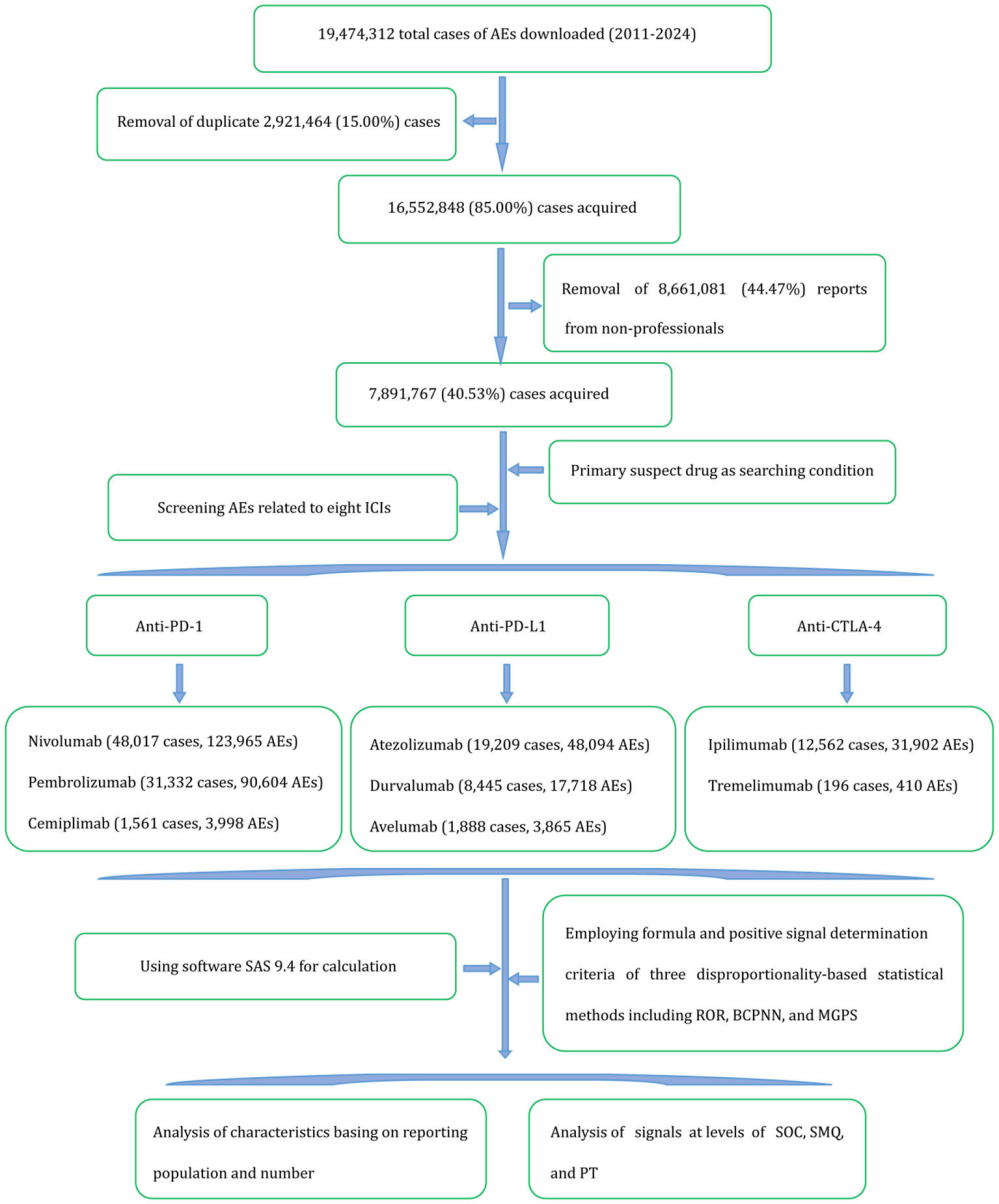
Flowchart for the data extraction and processing of adverse events associated with eight immune checkpoint inhibitors. AEs, adverse events; ROR, reporting odds ratio; BCPNN, Bayesian confidence propagation neural network; MGPS, multi-item gamma Poisson shrinker; SOC, System Organ Classification; SMQ, Standardized MedDRA Query; PT, Preferred Term.

All data in this study were obtained from the FAERS database. To ensure accuracy, a rigorous data extraction and cleaning process was implemented. First, duplicate reports were removed in accordance with FDA-recommended procedures. The duplicate removal algorithm was as follows (1): downloaded records were sorted by CASEID, FDA_DT (report date), and PRIMARYID. (2) for records sharing identical CASEID, the record with the maximum FDA_DT was retained, and earlier records were discarded. (3) for records with identical CASEID and FDA_DT, the record with the maximum PRIMARYID value was retained. (4) starting from 2019 Q1, records listed on the quarterly FDA deletion report were removed. Second, the identification of ICI-related irAEs requires professional expertise to accurately evaluate the potential influence of underlying conditions, metastases, paraneoplastic syndromes, pre-existing autoimmune diseases, and concomitant medications. To minimize reporting bias stemming from nonspecialized judgment, reports submitted by nonprofessionals, such as lawyers and consumers, were excluded. Third, AEs were screened using the primary suspect drug criterion, focusing on eight ICIs. Reporting characteristics and signal intensity were subsequently analyzed using appropriate statistical methods. The detailed data processing workflow is illustrated in **Figure 1**. Finally, all AEs at the PT level were categorized into 27 SOCs and 116 SMQs based on MedDRA version 27.1, with a narrow SMQ adopted.

### 2.2 Signal mining and statistical analysis

According to literatures (12, 13), the commonly used disproportionality-based statistical methods for post-marketing drug safety include the Reporting Odds Ratio (ROR), Proportional Reporting Ratio (PRR), Medicines and Healthcare products Regulatory Agency (MHRA), Bayesian confidence propagation neural network (BCPNN), and Multi-item gamma Poisson shrinker (MGPS). To preliminarily investigate the number of positive signals generated by different statistical analysis methods, we analyzed the reporting data for nivolumab and atezolizumab using these various statistical methods. The results from the small sample experiments are shown in **Supplementary Table 1**. Notably, during the small sample experiment, this study observed that for the same ICI, the positive signals derived from the ROR, PRR, and BCPNN methods were approximately equivalent. In contrast, the MGPS method, which employs a stringent threshold of the lower limit of 95% confidence interval of empirical Bayesian geometric mean (EBGM_05_) > 2, produced the fewest signals. The positive signals generated by the MHRA method fell in between these two groups. Given that this study aimed to investigate the clinical disease spectrum of irAEs associated with ICIs. Consequently, at the SOC level, both the ROR and BCPNN methods were utilized for mutual validation, the MGPS method was applied at the SMQ level, and the BCPNN method was employed at the PT level to identify strong positive signals for analysis, thereby enhancing the visibility of significant signals across various levels.

For all statistical methods, a minimum of three target reports was required as a basic condition. Additionally, the criteria for screening positive signals varied by method: the lower limit of 95% confidence interval of ROR (ROR_025_) >1 for ROR, the lower limit of 95% confidence interval of the information component (IC_025_) >0 for BCPNN, and the EBGM_05_ >2 for MGPS. The IC_025_ from the BCPNN method was further used to assess signal strength at hierarchical levels, such as PT and SOC. Signal intensity was classified as follows: 0 < IC_025_ ≤ 1.5 indicates a weak signal (+), 1.5 < IC_025_ ≤ 3.0 indicates a moderate signal (++), and IC_025_ > 3.0 indicates a strong signal (+++). Statistical analyses were performed using SAS version 9.4. The calculation formula and positive signal determination criteria are based on methods from relevant literatures (12, 13). Details are presented in **Supplementary Table 2**.

### 2.3 Descriptive analysis

Various figures and tables were utilized for multiple purposes. First, combined tables and figures summarized the characteristics of ICI-related AEs, including sex, age, reporting trends, time to onset, continent of reporting, reporter occupation, outcomes, and reporting type. Second, reporting quantity, proportion of death, and signal intensity at the SOC, SMQ, and PT levels were presented using combined figures, including butterfly diagrams, heatmaps, circle, Venn, and bar diagrams.

## 3 Results

### 3.1 Reporting population

Considering that ICIs—including ipilimumab, nivolumab, pembrolizumab, atezolizumab, avelumab, durvalumab, cemiplimab, and tremelimumab—were approached in 2011, 2014, 2014, 2016, 2017, 2017, 2018, and 2022, respectively, we retrieved FAERS reports from January 2011 to December 2024 to serve as the background database and conducted statistical analyses based on these data. A total of 19,474,312 reports were initially downloaded. After removing 2,924,464 duplicate records using CASEID and PRIMARYID matching, and excluding 8,661,081 reports submitted by nonprofessional reporters (e.g., consumers, lawyers, and unknown reporter types), 7,891,767 unique case reports remained for analysis. Using the primary suspected drug as the selection criterion, 320,556 AEs involving 123,210 cases associated with the eight ICIs were identified. The distribution of reports for each ICI is illustrated in **Figure 1**.

### 3.2 Reporting characteristics of ICI-related AEs

**Figure 2:**
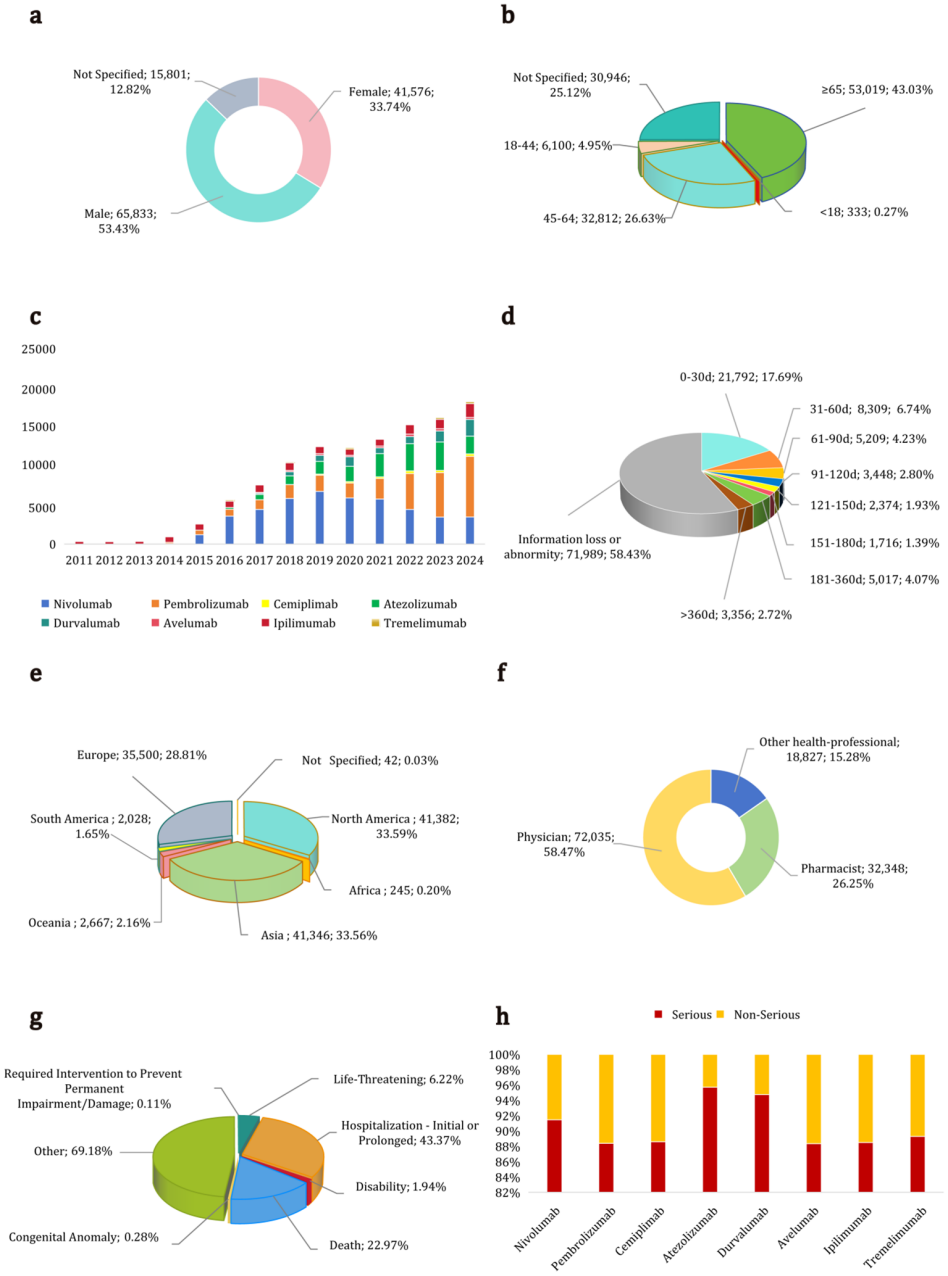
Reporting characteristics of adverse events associated with eight immune checkpoint inhibitors from 2011 to 2024. **(a)** Sex distribution of patients. **(b)** Age distribution of patients. **(c)** Reporting trends from 2011 to 2024. **(d)** Time to onset distribution. Time to onset is obtained by subtracting the start time of treatment from the start time of the event, and further summarized on a monthly basis. **(e)** Distribution of reporting countries by continent. **(f)** Occupation distribution of reporters. **(g)** Outcome distribution. **(h)** Distribution of reporting types.

**Figure 2**; **Supplementary Table 3** summarize the reporting characteristics of ICI-related AEs in the FAERS database from 2011 to 2024, presented as a composite graph and a detailed table, respectively. **Figures 2a, b** depict the sex and age distribution of the patients, respectively. Among the cases, 53.43% (65,833/123,210) were males, 33.74% (41,576/123,210) were females, and 12.83% (15,801/123,210) had unspecified sex. The median age for AEs associated with each ICI exceeded 65 years, with 57.46% (53,019/92,264) of patients aged over 65 among cases with complete age information, excluding 25.12% (30,946/123,210) with missing age data. **Figure 2c** illustrates the increasing trend in reported cases across the eight ICIs. Nivolumab, pembrolizumab, and atezolizumab collectively accounted for 79.99% (98,558/123,210) of the total cases, as presented in **Supplementary Table 3**. The distribution of the time to onset for ICI-related AEs is illustrated in **Figure 2d**. Among AE reports with documented time to onset information (n = 51,221; 41.57% of all reports), 68.94% (35,310/51,221) occurred within the first 3 months after initial ICI therapy in spite of few occurring one year later across all eight ICIs. This temporal pattern highlights the need for intensive monitoring during the first 3 months of ICI therapy, although AEs can emerge at any point during or after treatment. Median times to onset for individual ICIs ranged from 28 to 50 days, as detailed in **Supplementary Table 3**. **Figures 2e, f** show the distribution of reporters by continent and occupation, respectively. Asia, North America, and Europe were the top three continents, accounting for 95.96% (118,228/123,210) of the reports. After excluding reports from nonprofessionals, reports from physicians accounted for the highest proportion at 58.47% (72,035/123,210), followed by pharmacists at 26.25% (32,348/123,210), and other health professionals at 15.28% (18,827/123,210). **Figures 2g, h** illustrate the distribution of outcomes and reporting types for ICI-related AEs. Serious cases and deaths accounted for 91.17% (112,336/123,210) and 22.97% (28,307/123,210) of reports, respectively. The serious category included hospitalization, disability, required interventions, other severe outcomes, and death.

### 3.3 Distribution of reporting quantity, mortality risk, and signal intensity at the SOC level based on ROR method

The top 10 organic systems with the highest reporting frequency are as follows: general disorders and administration site conditions; gastrointestinal disorders; respiratory, thoracic, and mediastinal disorders; investigations; injury, poisoning, and procedural complications; benign, malignant, and unspecified neoplasms; infections and infestations; nervous system disorders; skin and subcutaneous tissue disorders; and metabolism and nutrition disorders. Nivolumab, pembrolizumab, and atezolizumab collectively accounted for 81.94% of all AEs. The distribution of the reporting frequency and proportion of ICI-related AEs by SOC is shown in **Supplementary Figure 1**.

**Figure 3:**
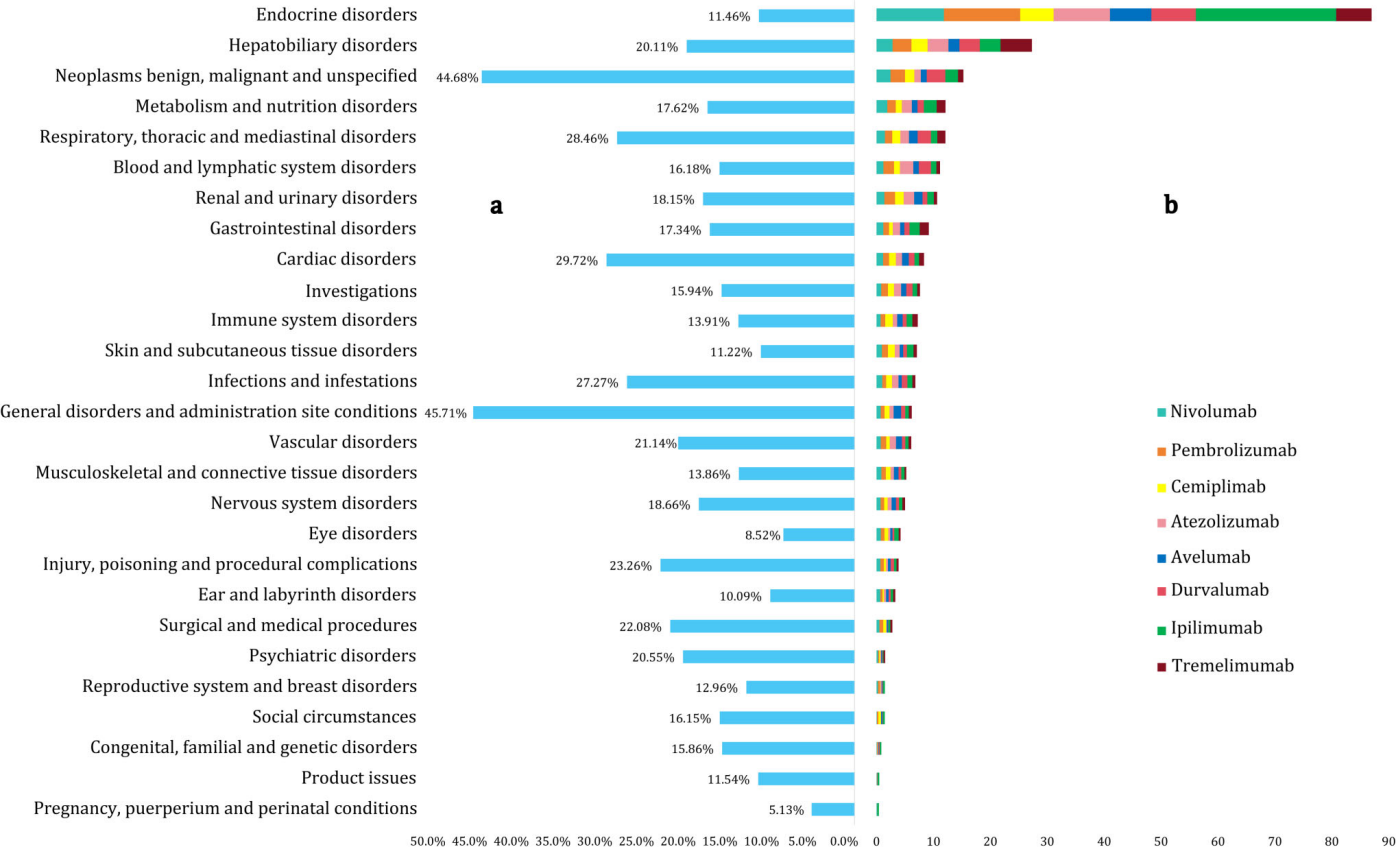
Proportion of deaths and cumulative ROR_025_ values for adverse events associated with eight immune checkpoint inhibitors by system organ classification from 2011 to 2024. **(a)** proportion of deaths. **(b)** cumulative ROR_025_. ROR_025_, lower limit of the 95% confidence interval for the Reporting Odds Ratio.

**Figures 3a, b** illustrates proportion of deaths, along with the corresponding the cumulative ROR_025_ (C-ROR_025_) values. The 5 organ systems most affected based on C-ROR_025_ values are as follows: endocrine disorders; hepatobiliary disorders; benign, malignant, and unspecified neoplasms; metabolism and nutrition disorders; respiratory, thoracic, and mediastinal disorders. Endocrine and hepatobiliary disorders were particularly prominent, ranking first and second in terms of C-ROR_025_ values, respectively.

Although the overall proportion of death cases was 22.97%, significant differences were observed among organ systems. The 5 organ systems with the highest proportion of deaths are as follows: general disorders and administration site conditions; benign, malignant, and unspecified neoplasms; cardiac disorders; respiratory, thoracic, and mediastinal disorders; infections and infestations. Excluding tumor-dependent conditions such as general disorders, tumor progression, various complications, and infections, irAEs that affect critical organ systems—including respiratory, cardiovascular, hepatic, and psychiatric systems—exhibited a high mortality risk.

Notably, although endocrine disorders had the highest C-ROR_025_ value, their associated proportion of death cases was not the highest. Conversely, cardiac disorders, which ranked ninth in signal strength, had the third-highest proportion of death. These findings indicate that signal strength and mortality risk are not directly correlated, highlighting substantial variability in the clinical impact of irAEs across different organ systems.

### 3.4 Statistical data of positive signals at the SOC level based on BCPNN method

**Figure 4:**
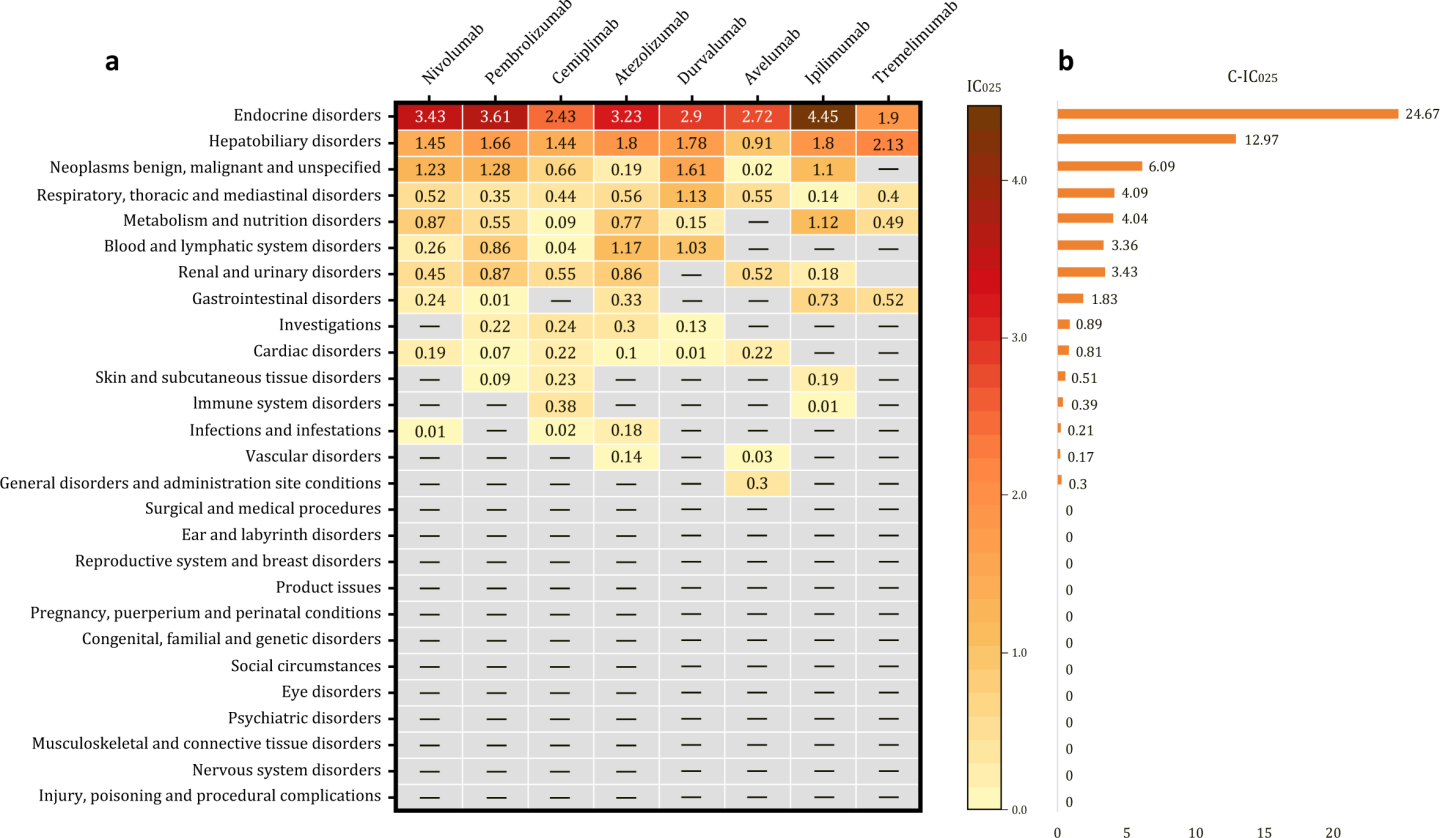
Positive signals associated with eight immune checkpoint inhibitors based on the BCPNN method at the SOC level. **(a)** Heatmap of positive signals and their lower limit of the 95% confidence interval for the information component (IC_025_) values. **(b)** Cumulative IC_025_ (C-IC_025_) values of positive organ systems. “—” indicates a negative signal.

Using the BCPNN method at the SOC level, 15 organ systems exhibited positive signals with IC_025_ values exceeding 0.0, whereas the remaining 12 systems showed negative signals, as illustrated in **Figures 4a, b**. Excluding tumor-dependent conditions, the top three signal-intense systems affected by irAEs were endocrine disorders, hepatobiliary disorders, and respiratory, thoracic, with cumulative IC_025_ (C-IC_025_) values of 24.67, 12.97, and 4.09, respectively (**Figure 4b**). These results are generally consistent with the findings obtained using the ROR method. Notably, four ICIs within the endocrine system—nivolumab, pembrolizumab, atezolizumab, and ipilimumab—exhibited strong positive signals, each with IC_025_ values exceeding 3.0. In the hepatobiliary system, five ICIs exhibited moderate signal intensity, with IC_025_ values ranging between 1.5 and 3.0. Signal intensity varied among different ICIs across organ systems (**Figure 4a**).

### 3.5 Statistical data of positive signals at the SMQ level based on MGPS method

**Figure 5:**
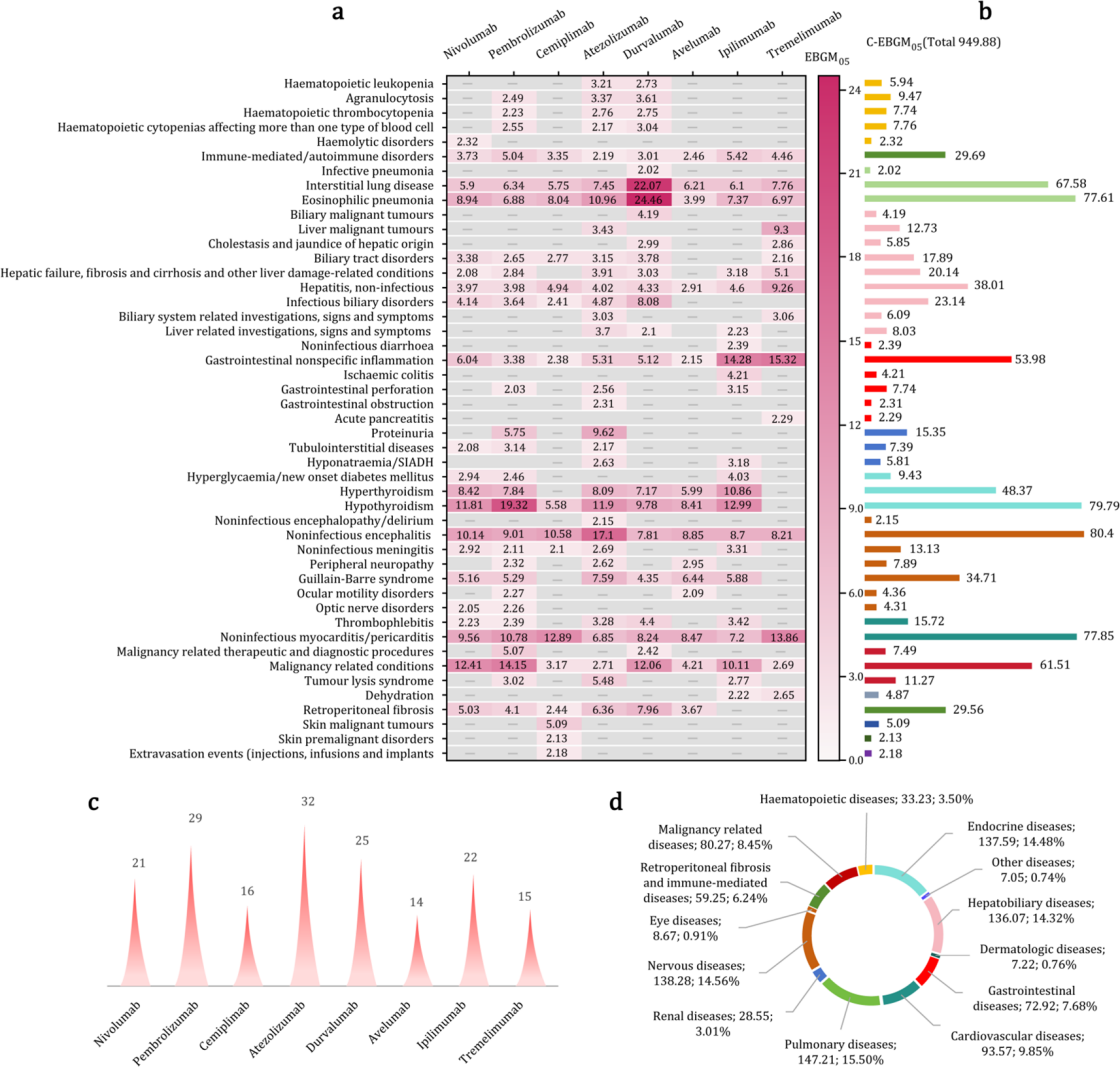
Positive signals associated with eight immune checkpoint inhibitors based on the MGPS method at the SMQ level. **(a)** Heatmap of positive signals and their lower limit of the 95% confidence interval for the empirical Bayesian geometric mean (EBGM_05_) values. **(b)** Cumulative EBGM_05_ (C-EBGM_05_) values of positive signals. **(c)** Numbers of positive signals of eight ICIs. **(d)** Distribution of C-EBGM_05_ values in vital organ systems. “—” indicates a negative signal.

To characterize the general disease classification of ICI-related AEs, the MGPS method was employed for positive signal detection at the SMQ level, and the results are illustrated in **Figures 5a–d**. The number of positive signals identified for each ICI is illustrated in **Figure 5c**. After integration, 47 of the 110 analyzed diseases exhibited positive signals. Detailed EBGM_05_ of diseases are presented in **Figure 5a**, with a cumulative EBGM_05_ (C-EBGM_05_) value of 949.88. The top 10 diseases ranked by C-EBGM_05_ values are illustrated in **Figure 5b**: noninfectious encephalitis, hypothyroidism, noninfectious myocarditis and pericarditis, eosinophilic pneumonia, interstitial lung disease, malignancy-related conditions, gastrointestinal nonspecific inflammation, hyperthyroidism, noninfectious hepatitis, and Guillain–Barré syndrome. These results suggest that irAEs often involve critical organs, including the brain, thyroid, heart, lungs, gastrointestinal tract, liver, and nerves. Additionally, irAEs affecting the hematologic system, kidneys, pancreas, skin, and eyes were also commonly observed. The distribution of C-EBGM_05_ values in vital organ systems is illustrated in **Figure 5d**.

### 3.6 Strong positive signals at the PT level based on the BCPNN method

**Figure 6:**
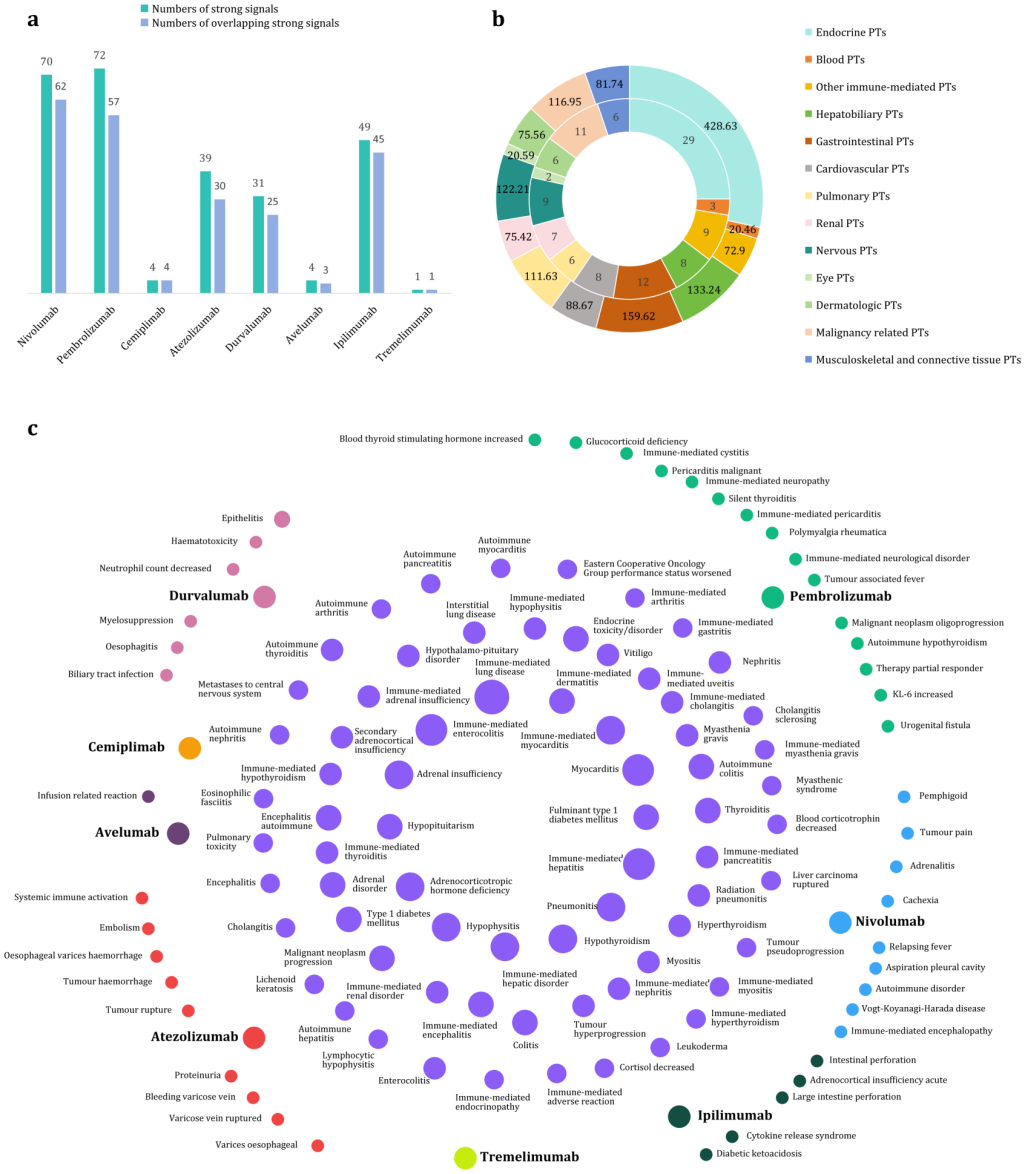
Strong positive signals associated with eight immune checkpoint inhibitors based on the BCPNN method at the PT level. **(a)** Numbers of strong signals and the overlapping strong signals for eight immune checkpoint inhibitors. **(b)** Numbers of strong signals in inner ring and their cumulative IC_025_ values in outer ring across vital organ systems. **(c)** Venn diagram of strong positive signals and their overlapping. The purple dots indicate overlapping strong signals, while the dots in other colors represent unique strong signals of different immune checkpoint inhibitors.

Strong positive signals associated with ICIs were identified at the PT level using the BCPNN method, in accordance with threshold criteria established in the literature (13). A total of 70, 72, 4, 39, 31, 4, 49, and 1 strong positive signals were detected for nivolumab, pembrolizumab, cemiplimab, atezolizumab, avelumab, durvalumab, ipilimumab, and tremelimumab, respectively (**Figure 6a**). After integrating these signals, 116 PTs were identified as exhibiting strong positive signals, with a cumulative C-IC_025_ value of 1507.63. The distribution of strong positive PT numbers, along with their C-IC_025_ values across vital organ systems, is illustrated in **Figure 6b**. Among 116 PTs, 71 overlapping strong positive PTs were shared across different ICIs (**Figure 6c**).

**Figure 7:**
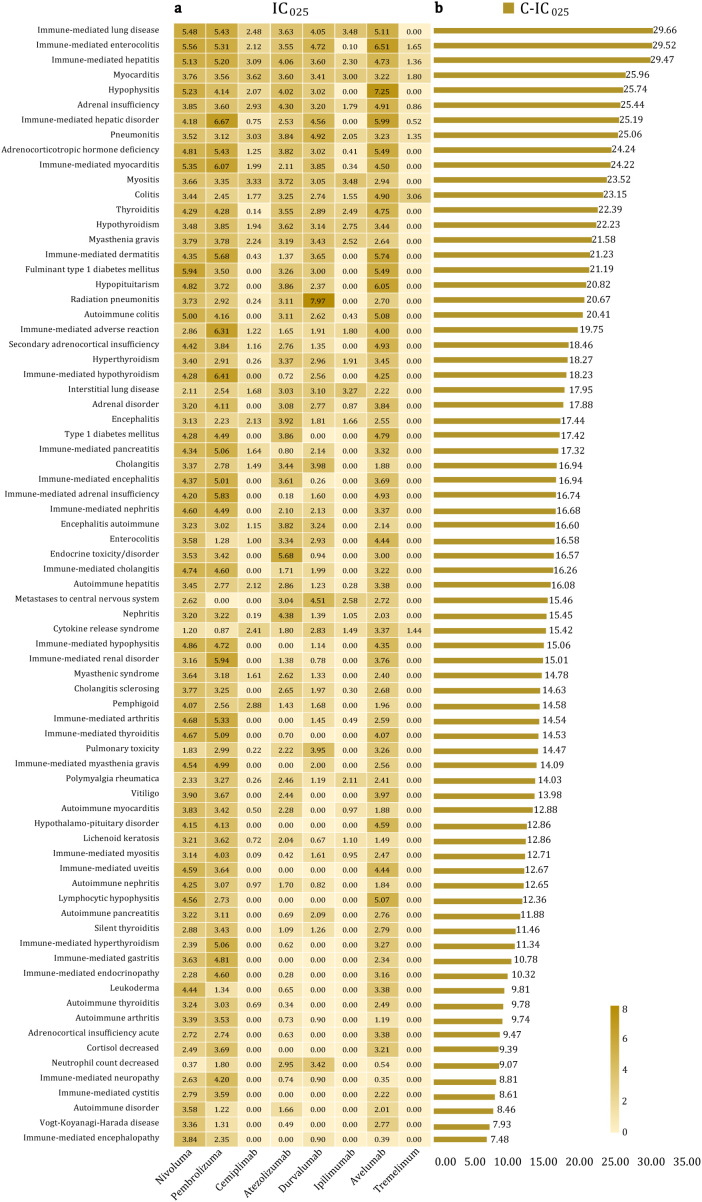
Detailed statistical data on the IC_025_ values for the eight immune checkpoint inhibitors and the C-IC_025_ values for the top 75 tumor-independent strong positive PTs. **(a)** IC_025_ values. **(b)** C-IC_025_ values.

Detailed statistical data on the IC_025_ values for the eight ICIs and C-IC_025_ values for the top 75 tumor-independent PTs are presented in **Figures 7a, b**, respectively. The top 10 PTs and their corresponding C-IC_025_ values were immune-mediated lung disease, immune-mediated enterocolitis, immune-mediated hepatitis, myocarditis, hypophysitis, adrenal insufficiency, immune-mediated hepatic disorder, pneumonitis, adrenocorticotropic hormone deficiency, and immune-mediated myocarditis. Additional PTs with significant signals included myositis, colitis, thyroiditis, immune-mediated dermatitis, hypopituitarism, myasthenia gravis, hypothyroidism, and fulminant type 1 diabetes mellitus.

## 4 Discussion

Although ICIs have brought transformative advances to the treatment of multiple cancers, their associated irAEs can affect nearly all organ systems. Symptoms of ICI-related irAEs are often nonspecific and may overlap with cancer-related manifestations or complications arising from other therapies. This overlap underscores the need for substantial clinical expertise for prompt recognition and accurate diagnosis. Compared with other AEs, the assessment of irAEs is complicated by a greater number of influencing factors, requiring a broader and more specialized knowledge base. The aim of this study is to provide a reference framework to support professional collaboration in the management of irAEs. Although excluding reports submitted by nonprofessionals may reduce data completeness, it strengthens the robustness and reliability of the findings by focusing on 14 years of systematically collected data. To date, this study represents the first pharmacovigilance analysis to evaluate the full spectrum of 320,556 ICI-related AEs using large-scale real-world data from the FAERS database spanning 2011 to 2024. A key contribution of this study lies in its multidimensional characterization of signals and their variability across different analytical levels, including PT, SMQ, and SOC. This integrated approach enables a more accurate and efficient understanding of irAEs associated with ICIs. In this analysis, the ratio of AEs to cases was 2.60:1 (320,556/123,210), indicating that individual patients may experience multiple AEs, a finding consistent with real-world clinical observations. For instance, a previously reported case described a 60-year-old man with disseminated metastatic melanoma who developed immune-mediated uveitis/retinitis and nephritis following the third dose of ipilimumab (14).

This study indicates that male patients have a higher risk of developing ICI-related AEs than female patients. This sex-based difference is consistent with findings from the World Health Organization (WHO) pharmacovigilance database (VigiBase) (15). Additionally, older patients exhibited a higher risk of ICI-related AEs than younger individuals, a pattern that aligns with previous research on age-related susceptibility to irAEs (16). The high proportion of older patients observed in this study can be attributed to the following factors: (1) the higher incidence of cancer in older adults results in a greater number of patients receiving ICIs; and (2) real-world evidence suggests that older oncology patients are more likely to experience severe and multiple irAEs than younger patients (16), consistent with the high proportion of severe ICI-related AEs (91.17%) observed in this analysis.

At the SOC level, statistical analysis indicates that the proportion of reported AEs in each organ system does not directly correlate with signal intensity or mortality risk. Excluding various potentially tumor-dependent conditions, the organ systems with the highest and second-highest numbers of reported irAEs are the gastrointestinal disorders and respiratory, thoracic, and mediastinal disorders. However, the organ systems associated with the highest mortality risk are cardiac disorders and respiratory, thoracic, and mediastinal disorders. This observation is generally consistent with data from the WHO Vigibase regarding fatal ICI-related AEs (17). Across both the ROR and BCPNN methods, endocrine disorders and hepatobiliary disorders consistently exhibit the highest signal intensities. Comprehensive understanding of organ systems with a high mortality risk and strong signal intensity is crucial, as timely identification and management of irAEs in these critical systems are essential for enhancing patient quality of life and prolonging survival during cancer therapy.

Endocrine irAEs represent the most prominent signals associated with ICIs, exhibiting the highest C-ROR_025_ and C-IC_025_ values at the SOC level (**Figures 3b**, **4b**). Using the MGPS method, three corresponding SMQs—hyperglycemia/new-onset diabetes mellitus, hyperthyroidism, and hypothyroidism—were identified, accounting for 14.48% of the C-EBGM_05_ values (137.59/949.88) (**Figure 5b**). Through strong-signal screening using the BCPNN method, 29 PTs with IC_025_ values >3 were identified, representing 25.00% (29/116) (**Figure 6b**) of all strong positive signals. The top 10 overlapping PTs based on C-IC_025_ were hypophysitis, adrenal insufficiency, adrenocorticotropic hormone deficiency, hypopituitarism, thyroiditis, immune-mediated adrenal insufficiency, fulminant type 1 diabetes mellitus, (immune-mediated) hypothyroidism, secondary adrenocortical insufficiency, and hyperthyroidism (**Figures 6c**, **7b**). These findings indicate that the pituitary, adrenal, thyroid, and pancreas are frequently affected by immune dysregulation, consistent with recent research findings (18). Signals for hypophysitis, adrenocorticotropic hormone deficiency, and adrenal insufficiency were found to be stronger than those for hypothyroidism and hyperthyroidism. This differs somewhat from previous studies (4, 19, 20), likely due to methodological differences. Specifically, the conclusion in reference (4) was based on a comparison of AE counts, whereas references (19) and (20) conducted meta-analyses of clinical trial data. In contrast, the present study relies on disproportionality analysis of spontaneous reporting system data, underscoring how variations in analytical approach and data context can influence outcomes. Given that multigland involvement is more likely during combination immunotherapy (18, 21), and that dysfunction in one endocrine gland can cascade into others—for example, adrenal insufficiency may result from hypophysitis, which can be life-threatening—a comprehensive evaluation of hormonal axis interdependence is essential, and special care should be provided for patients receiving combination ICIs. Our analysis further indicates that hypophysitis and hypopituitarism are strongly associated with the anti-CTLA-4 agent ipilimumab (IC_025_ = 7.25 and 6.05, respectively **Figure 7a**), whereas immune-mediated (hyperthyroidism and hypothyroidism) are strongly associated with the anti-PD-1 agent pembrolizumab (IC_025_ = 5.06 and 6.41, respectively, **Figure 7a**), consistent with previous reports (18, 21). ICI-induced endocrinopathies are typically chronic rather than fatal, with a mortality rate ranking of 23rd; however, they may result in permanent endocrine dysfunction, necessitating lifelong hormone replacement therapy to maintain patient quality of life.

Hepatobiliary disorders are the second most common irAEs after endocrine toxicities at the SOC level (**Figures 3b**, **4b**). They exhibited a spectrum of nine positive signals at the SMQ level and eight strong PT manifestations. In comparison, gastrointestinal disorders showed six positive signals at the SMQ level and 12 strong positive PT manifestations (**Figures 5b**, **6b**). Collectively, hepatobiliary and gastrointestinal signals accounted for 31.91% (15/47) of positive SMQ-level signals, with a C-EBGM_05_ value of 22.00% (208.99/949.88) (**Figure 5d**). Nonspecific gastrointestinal inflammation and noninfectious hepatitis ranked seventh and ninth, respectively, among the 47 positive SMQ diseases (**Figure 5b**). Common PTs of hepatobiliary toxicities include immune-mediated hepatitis (hepatic disorder) and (immune-mediated) cholangitis, whereas gastrointestinal toxicities commonly present as (immune-mediated) enterocolitis, (autoimmune) colitis, immune-mediated pancreatitis, and immune-mediated gastritis, among others (**Figures 6c**, **7b**). These findings are consistent with previous reviews (22). ICI-mediated pancreatitis is characterized by autoimmune injury of the exocrine pancreas resulting from ICI exposure (23). Digestive system irAEs are heterogeneous in presentation. Treatment options range from symptomatic management to temporary withholding ICI doses, or permanent discontinuation. The need for immunosuppressive therapy with corticosteroids or alternative agents is guided by various grades of irAEs and their clinical resolution (24–26). In steroid-refractory cases, biological therapies—such as IL-12/23 blockade with ustekinumab and IL-6 blockade with tocilizumab—have shown clinical efficacy (27, 28).

Cardiotoxicity carries a higher mortality risk than vascular toxicity at the SOC level (**Figure 3a**). Using the MGPS method at the SMQ level, thrombophlebitis and noninfectious myocarditis/pericarditis were identified as key cardiovascular signals. Their combined C-EBGM_05_ values accounted for 9.85% (93.57/949.88), with noninfectious myocarditis/pericarditis ranking third among the 47 positive SMQs (**Figures 5b, d**). At the PT level, the BCPNN method identified eight strong positive signals, including autoimmune or immune-mediated myocarditis, immune-mediated pericarditis, malignant pericarditis, embolism, and ruptured or bleeding varicose veins, with (immune-mediated) myocarditis showing the highest overlap (**Figures 6b, c**, **7a**). This study found that ICI-related cardiovascular toxicity can also manifest as arrhythmia, vasculitis, thromboembolism, heart failure, and may potentially accelerate coronary atherosclerosis, presenting with moderate or weak signal. These clinical manifestations align with current authoritative statements and scientific reviews (29–31). ICI-associated myocarditis may coexist with myositis or myasthenia gravis, conditions that can be severe and indicate a poor prognosis, particularly when accompanied by cardiogenic shock, heart block, or incessant ventricular arrhythmias, often resulting in fatal outcomes (31–34).

Respiratory and renal toxicities associated with ICIs ranked fourth and seventh, respectively, at the SOC level (**Figure 4b**). At the SMQ level, pulmonary toxicity, which manifests as eosinophilic pneumonia, interstitial lung disease, and infective pneumonia, exhibited a combined C-EBGM_05_ of 15.50% (147.21/949.88), significantly surpassing renal toxicity, which is characterized by proteinuria, tubulointerstitial diseases, and hyponatremia/SIADH, with a combined C-EBGM_05_ value of 3.00% (28.55/949.88) (**Figure 5d**). ICIs, particularly when combined with chemotherapy, antibody–drug conjugates, or targeted therapy, are significant contributors to drug-induced pneumonitis during systemic therapy for lung cancer (35, 36). At the PT level, the most prominent overlapping signals included immune-mediated lung disease, pneumonitis, interstitial lung disease, (immune-mediated) nephritis, and renal disorders (**Figure 6c**, **7b**). Although both chemotherapy and ICIs can cause renal injury, ICI-related renal toxicity predominantly manifests as acute tubulointerstitial nephritis, whereas chemotherapy-related renal injury typically presents as acute tubular injury or necrosis. Accurately distinguishing the cause of renal injury in patients receiving concurrent ICI and chemotherapy is essential, as it informs decisions regarding which therapy can be safely continued and which should be discontinued (37, 38).

Although various ICIs generally exhibited negative signals for nervous system and eye disorders at the SOC level, seven positive signals (7/47, 14.89%) were identified at the SMQ level. Using the MGPS method, these signals accounted for 15.47% of the C-EBGM_05_ value (146.95/949.88) (**Figures 5b, d**). Among the 47 positive SMQs analyzed, noninfectious encephalitis and Guillain–Barré syndrome ranked first and tenth, respectively (**Figure 5b**). At the PT level, 11 strong signals were identified within the nervous system and eye disorders, with (immune-mediated) encephalitis, (immune-mediated) myasthenia gravis, and autoimmune encephalitis showing significant overlap (**Figures 6c**, **7**). Previous research has reported that ICI-related ophthalmic irAEs may involve the anterior and posterior segments and ocular nerves, presenting as ophthalmoplegia, uveitis, dry eye, conjunctivitis, blepharitis, and keratitis (39, 40). Consistent with findings from VigiBase (15), this analysis identified not only immune-mediated uveitis but also Vogt–Koyanagi–Harada disease as exhibiting strong positive signals (**Figures 6c**, **7**). Ophthalmoplegia associated with ICIs is a recognized complication of myasthenia gravis (40). Patients with myasthenia gravis face an elevated risk of respiratory suppression and myocarditis, both of which can be life-threatening. Given the high mortality of severe myasthenia gravis in lung cancer patients, comprehensive monitoring and vigilant clinical management are essential for this population.

In addition to the ICI-related irAEs affecting the previously discussed vital organ systems, rheumatic (musculoskeletal and connective) and dermatologic disorders warrant particular attention. These irAEs can impair the normal function of muscles, bones, connective tissues, and the skin, with C-IC_025_ of PT signals accounting for approximately 10.43% (157.30/1507.63) of the total (**Figure 6b**). Most of these disorders are chronic and frequently encountered in clinical practice, presenting with a broad spectrum of symptom. Common manifestations include immune-mediated myositis, arthritis, and dermatitis, as well as polymyalgia rheumatica, vitiligo, and leukoderma (**Figures 6c**, **7**). Although the mortality risk associated with most rheumatic and skin disorders is lower than that of other organ system toxicities, the associated pain, functional limitation, and visible skin changes can significantly compromise patients’ quality of life, leading to substantial physical, psychological, and social burden. Improved outcomes may be achieved through increased clinical awareness, multidisciplinary management, and enhanced social support. Notably, ICI-related myositis, particularly when occurring concurrently with myocarditis and myasthenia gravis, is associated with a marked increase in mortality risk (41).

### 4.1 Mechanisms of ICI toxicities

The pathophysiological mechanisms underlying irAEs across different organ systems are heterogeneous and multifactorial, rather than driven by a single common pathway. Several hypotheses have been proposed to explain these toxicities. First, ICIs may induce aberrant activation of self-reactive T cells, leading to immune-mediated damage of noncancerous tissues expressing PD-L1, such as pancreatic β cells, renal tubular epithelial cells, and hepatocytes (18, 37, 42), or those associated with CTLA-4, including the myocardium and pituitary glands (18, 29). This dysregulated activation triggers inflammatory responses and immune cell infiltration, involving CD8+ T lymphocytes, monocytes, and macrophages. These processes lead to off-target organ toxicities or functional impairments, clinically manifesting as liver injury, acute tubulointerstitial nephritis, myocarditis, hypophysitis, and diabetes mellitus (18, 29, 37, 42). Second, ICIs may disrupt peripheral immune tolerance, promoting the production of autoantibodies directed against tissue-specific antigens, such as cardiac α-myosin or renal-specific tubular antigens (29, 37). Third, the reactivation of drug-specific autoreactive T cells may contribute to ICI-associated acute kidney injury. Many cancer patients receive concomitant medications, such as proton pump inhibitors and nonsteroidal anti-inflammatory drugs, which can bind to renal tubular antigens and act as haptens. ICIs may amplify these hapten-induced immune responses, resulting in tubular injury (37). Fourth, shared antigens between tumor cells and inflamed tissues can promote cross-reactive immune responses. One study identified nine T-cell antigens shared between tumor tissue and skin in patients with ICI-related dermatitis, supporting this hypothesis (43). Fifth, environmental and infectious triggers may influence irAE development. For example, specific strains of microbial flora colonization have been associated with ICI-related colitis (44), while Epstein–Barr virus infection may contribute to ICI-related encephalitis (45). Sixth, pre-existing, smoldering inflammation can be exacerbated by ICI treatment. For instance, patients with baseline autoantibodies, such as antithyroid antibodies, are at higher risk of developing thyroid dysfunction after ICI therapy compared with those without such antibodies (18). Finally, dysregulated cytokine signaling plays critical in the pathogenesis of irAEs. Proinflammatory cytokines, including tumor necrosis factor (TNF), interleukin (IL)-1, IL-6, and IL-17, have been implicated. Elevated levels of IL-17A were reported in a patient who developed fatal neuroendocrine toxicity characterized by insulin-dependent diabetes, hypophysitis, and a myasthenia-like syndrome (46). Preclinical study has shown that TNF inhibitors, when used alongside anti-CTLA-4 and anti-PD-1 immunotherapy, can ameliorate colitis while enhancing antitumor efficacy (47). Obese mice exposed to ICIs and chemotherapy developed cardiac irAEs and exhibited elevated serum IL-1β levels (48). Clinical research has further shown that elevated serum IL-6 levels are associated with increased irAE incidence and poorer prognosis in patients receiving ICI therapy (49).

An in-depth understanding of the underlying mechanisms can provide valuable insights for improving the management of ICI-related irAEs. Clinical studies have further reported that IL-6 inhibitors (e.g., tocilizumab) and TNF inhibitors (e.g., infliximab, certolizumab) demonstrate promising clinical efficacy and an acceptable safety profile in the treatment of ICI-induced irAEs (27, 50).

### 4.2 Limitations

Several limitations of this study should be acknowledged. First, the use of the primary suspected drug as the screening criterion does not fully account for the impact of concomitant medications when multiple drugs are implicated. Second, given that FAERS is a spontaneous reporting system with global coverage, incomplete reporting is inevitable, preventing a precise estimation of the true incidence of ICI-related irAEs. Third, the purpose of this study was to characterize the overall spectrum of irAEs, using relatively stringent statistical methods and threshold criteria to identify important positive signals; however, this approach may have inadvertently excluded weaker signals. Fourth, differences in the market availability and prescribing patterns of ICIs across regions and time periods may introduce regional and temporal biases, potentially affecting the distribution of detected signals. More broadly, the FAERS database lacks detailed information on patients’ baseline comorbidities and treatment histories and is inherently subject to underreporting, reporting bias, outcome misclassification, and confounding related to indications and concomitant medications. Consequently, it cannot establish causality for ICI-related irAEs. Therefore, the results of this study should be interpreted as statistically significant signals rather than definitive evidence of causal relationships.

## 5 Conclusions

This study examines the demographic characteristics and associated risks of ICI-related irAEs using statistical methods. It highlights the heterogeneous clinical manifestations of irAEs across multiple organ systems and underscores the importance of timely recognition through interdisciplinary collaboration among oncologists and specialists such as endocrinologists, cardiologists, rheumatologists, and gastroenterologists. Early identification and management can significantly improve patients’ well-being and clinical outcomes. Key findings include (1) strongest signals in endocrine toxicities, such as hypophysitis, adrenocorticotropic hormone deficiency, fulminant type 1 diabetes mellitus, and hypothyroidism; (2) prominent signals in digestive, cardiac, and respiratory toxicities, including immune-mediated hepatitis, enterocolitis, lung disease, and myocarditis/pericarditis; (3) high mortality risks in cardiac and respiratory toxicities; and (4) other irAEs involving the nervous, renal, ocular, dermal, articular, and muscular systems. 

## Data Availability

The raw data supporting the conclusions of this article will be made available by the authors, without undue reservation.

## Glossary

AEs: Adverse events
anti-CTLA-4: Cytotoxic T-lymphocyte-associated antigen-4 inhibitors
anti-PD-1: Programmed death-1 inhibitors
anti-PD-L1: Programmed death-ligand 1 inhibitors
BCPNN: Bayesian confidence propagation neural network
C-EBGM_05_: Cumulative EBGM_05_
C-IC_025_: Cumulative IC_025_
C-ROR_025_: Cumulative ROR_025_
EBGM: Empirical Bayesian geometric mean
EBGM_05_: Lower limit of 95% confidence interval of empirical Bayesian geometric mean
IC: Information component
IC_025_(IC-2SD): Lower limit of 95% confidence interval of the information component
ICIs: Immune checkpoint inhibitors
irAEs: Immune-related adverse events
MedDRA: Medical Dictionary for Regulatory Activities
MGPS: Multi-item gamma Poisson shrinker
PT: Preferred Term
ROR: Reporting Odds Ratio
ROR_025_: Lower limit of 95% confidence interval of ROR
SMQ: Standardized MedDRA query
SOC: System Organ Classification.
