# Supplementary material for: Comprehensive safety analysis of the clinical spectrum of adverse events associated with immune checkpoint inhibitors based on FAERS
Source: Front Immunol. 2026 May 1;17:1823743. doi: 10.3389/fimmu.2026.1823743 (PMC13175965; doi:10.3389/fimmu.2026.1823743)
Supplement: Supplementary file 1 [file DataSheet1.docx]

Supplementary Material

# Supplementary Figure

**Supplementary Figure 1.** Number of adverse events and their proportions for the eight immune checkpoint inhibitors categorized by System Organ Classification in FAERS from 2011 to 2024. (a) Proportion distribution. (b) Reporting number.


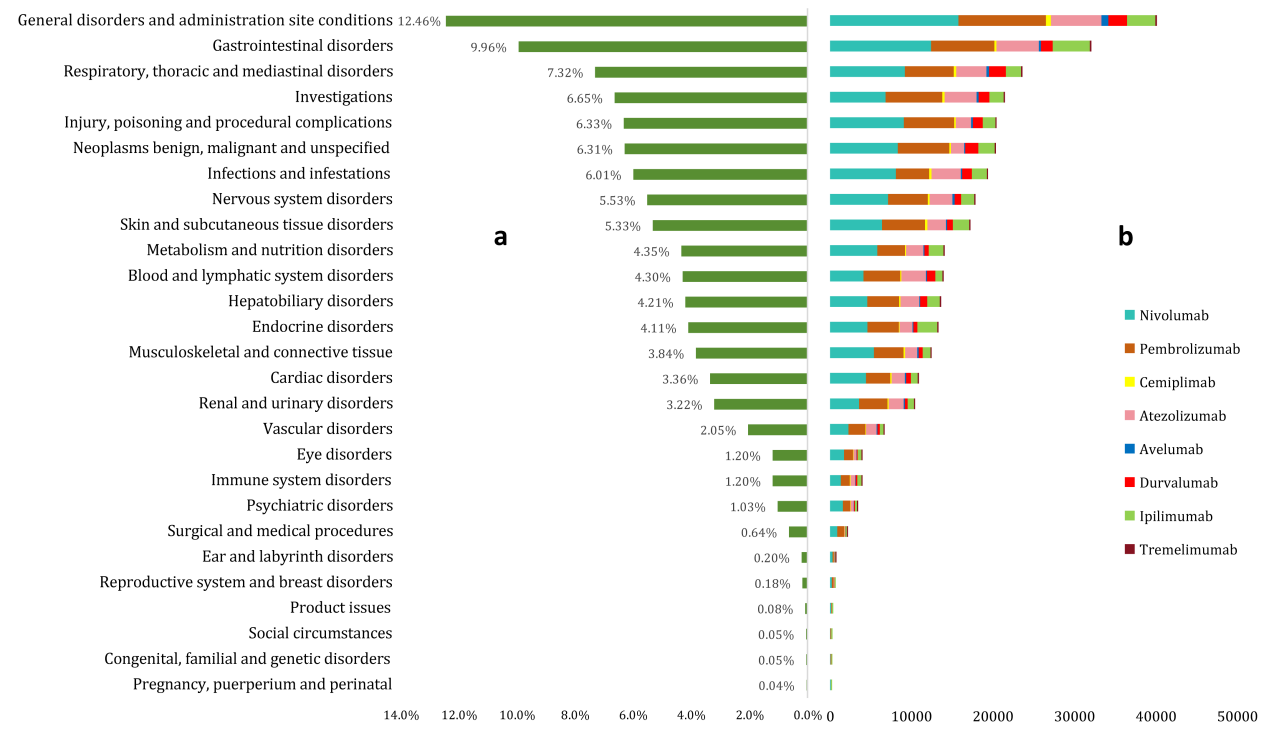


# **Supplementary Tables**

**2.1 Supplementary Table 1.** Small sample experiment results (Positive signal counts at at different levels using different statistical methods).

|  | **Method/Level** | **ROR** | **PRR** | **MHRA** | **BCPNN** | **MGPS** |
| --- | --- | --- | --- | --- | --- | --- |
| Nivolumab | SOC | 10 | 10 | 3 | 10 | 3 |
|  | SMQ | 58 | 58 | 25 | 58 | 21 |
|  | PT | 836 | 836 | 706 | 752 | 579 |
| Atezolizumab | SOC | 10 | 10 | 3 | 9 | 2 |
|  | SMQ | 66 | 66 | 38 | 66 | 34 |
|  | PT | 569 | 569 | 504 | 510 | 492 |

Notes

**ROR:** reporting odds ratio

**PRR:** proportional reporting ratio

**MHRA:** Medicines and Healthcare products Regulatory Agency

**BCPNN:** Bayesian confidence propagation neural network

**MGPS:** Multi-item gamma Poisson shrinker

**SOC:** System Organ Classification

**SMQ:** Standardized MedDRA Query

**PT:** Preferred Term

**2.2 Supplementary Table 2.**  Calculation formula and criteria of ROR, BCPNN, and MGPS methods.

| **Method** | **Calculation formula** | **Criteria** |
| --- | --- | --- |
| ROR | $ROR=\frac{(a/c)}{(b/d)}=\frac{ad}{bc}$  $SE(lnROR)=\sqrt{\left( \frac{1}{a}+\frac{1}{b}+\frac{1}{c}+\frac{1}{d} \right)}$  $95\%CI=e^{ln(ROR)\pm1.96\sqrt{\left( \frac{1}{a}+\frac{1}{b}+\frac{1}{c}+\frac{1}{d} \right)}}$ | $a\geq3$ and $\mathrm{ROR}$_025_ $>1$ |
| BCPNN | $IC=\log_{2}\frac{p(x,y)}{p(x)p(y)}=\log_{2}\frac{a(a+b+c+d)}{(a+b)(a+c)}$  $E(IC)=\log_{2}\frac{(a+\gamma11)(a+b+c+d+\alpha)(a+b+c+d+\beta)}{(a+b+c+d+\gamma)(a+b+\alpha1)(a+c+\beta1)}$  $V(IC)=\frac{1}{(ln2)^{2}}\left\{ \left[ \frac{(a+b+c+d)-a+\gamma-\gamma11}{(a+\gamma11)(1+a+b+c+d+\gamma)} \right]+\left[ \frac{(a+b+c+d)-(a+b)+\alpha-\alpha1}{(a+b+\alpha1)(1+a+b+c+d+\alpha)} \right]+\left[ \frac{(a+b+c+d)-(a+c)+\beta-\beta1}{(a+c+\beta1)(1+a+b+c+d+\beta)} \right] \right\}$  $\gamma=\gamma11\frac{(a+b+c+d+\alpha)(a+b+c+d+\beta)}{(a+b+\alpha1)(a+c+\beta1)}$  $IC-2SD=E(IC)-2\sqrt{V(IC)}$ | $a\geq3$ and IC_025_ $>0$；   1. 0 < IC_025_ ≤ 1.5: weak signal (+)；   （2）1.5 < IC_025_ ≤ 3.0: moderate signal (++)；  （3）IC_025_ > 3.0: strong signal (+++) |
| MGPS | $\text{EBGM}=\frac{a(a+b+c+d)}{(a+c)(a+b)}$  $95\%\text{CI}=e^{ln(\text{EBGM})\pm1.96\sqrt{\left( \frac{1}{2}+\frac{1}{b}+\frac{1}{c}+\frac{1}{d} \right)}}$ | $a\geq3$ and EBGM_05_ $>2$ |

Notes

**a:** the number of target drug induced target AE; **b:** the number of target drug induced other AE;

**c:** the number of other drugs induced target AE; **d:** the number of other drugs induced other AE;

**ROR:** reporting odds ratio; **IC:** information component; **EBGM:** empirical Bayesian geometric mean

**2.3 Supplementary Table 3.** Demographic data of adverse drug events associated with eight immune checkpoint inhibitors from 2011 to 2024

| **Maker** | **Nivolumab** | **Pembrolizumab** | **Cemiplimab** | **Atezolizumab** | **Durvalumab** | **Avelumab** | **Ipilimumab** | **Tremelimumab** | |
| --- | --- | --- | --- | --- | --- | --- | --- | --- | --- |
| **Sex distribution of patients** | | | | | | | | |  |
| Female (%) | 14402 (29.99) | 14209 (45.35) | 321 (20.56) | 6538 (34.04) | 2134 (25.27) | 490 (25.95) | 3445 (27.42) | 37 (18.88) |  |
| Male (%) | 27045 (56.32) | 15625 (49.87) | 498 (31.90) | 10094 (52.55) | 4419 (52.33) | 1204 (63.77) | 6856 (54.58) | 92 (46.94) |  |
| Not Specified (%) | 6570 (13.68) | 1498 (4.78) | 742 (47.53) | 2577 (13.42) | 1892 (22.40) | 194 (10.28) | 2261 (18.00) | 67 (34.18) |  |
| **Age distribution of patients** | | | | | | | | |  |
| <18 (%) | 216 (0.45) | 68 (0.22) | 2 (0.13) | 21 (0.11) | 2 (0.02) | 2 (0.11) | 22 (0.18) | 0 (0.00) |  |
| 18-44 (%) | 2520 (5.25) | 1703 (5.44) | 19 (1.22) | 885 (4.61) | 147 (1.74) | 42 (2.22) | 781 (6.22) | 3 (1.53) |  |
| 45-64 (%) | 13186 (27.46) | 8563 (27.33) | 145 (9.29) | 5160 (26.86) | 1943 (23.01) | 430 (22.78) | 3343 (26.61) | 42 (21.43) |  |
| ≥65 (%) | 19641 (40.90) | 14303 (45.65) | 376 (24.09) | 9082 (47.28) | 3672 (43.48) | 989 (52.38) | 4894 (38.96) | 62 (31.63) |  |
| Not Specified (%) | 12454 (25.94) | 6695 (21.37) | 1019 (65.28) | 4061 (21.14) | 2681 (31.75) | 425 (22.51) | 3522 (28.04) | 89 (45.41) |  |
| Median  (Q1,Q3) | 66.00  (57.00,73.00) | 67.00  (58.00,75.00) | 71.00  (62.00,79.00) | 68.00  (59.00,74.00) | 68.00  (61.00,74.00) | 70.00  (61.00,77.00) | 66.00  (56.00,73.00) | 67.00  (58.00,74.00) |  |
| **Reporting quantity by year** | | | | | | | | |  |
| 2011 (%) | 0 | 0 | 0 | 0 | 0 | 0 | 171 (1.36) | 0 |  |
| 2012 (%) | 0 | 0 | 0 | 0 | 0 | 0 | 337 (2.68) | 0 |  |
| 2013 (%) | 12 (0.02) | 2 (0.01) | 0 | 0 | 0 | 0 | 359 (2.86) | 0 |  |
| 2014 (%) | 66 (0.14) | 177 (0.56) | 0 | 0 | 2 (0.02) | 0 | 748 (5.95) | 0 |  |
| 2015 (%) | 1287 (2.68) | 625 (1.99) | 0 | 0 | 0 | 2 (0.11) | 827 (6.58) | 0 |  |
| 2016 (%) | 3834 (7.98) | 931 (2.97) | 0 | 233 (1.21) | 3 (0.04) | 2 (0.11) | 870 (6.93) | 1 (0.51) |  |
| 2017 (%) | 4744 (9.88) | 1322 (4.22) | 0 | 723 (3.76) | 172 (2.04) | 104 (5.51) | 1022 (8.14) | 0 |  |
| 2018 (%) | 6237 (12.99) | 1896 (6.05) | 16 (1.02) | 1185 (6.17) | 558 (6.61) | 187 (9.90) | 1036 (8.25) | 1 (0.51) |  |
| 2019 (%) | 7212 (15.02) | 2216 (7.07) | 148 (9.48) | 1751 (9.12) | 823 (9.75) | 230 (12.18) | 958 (7.63) | 0 |  |
| 2020 (%) | 6324 (13.17) | 2033 (6.49) | 190 (12.17) | 2089 (10.88) | 1321 (15.64) | 200 (10.59) | 862 (6.86) | 33 (16.84) |  |
| 2021 (%) | 6159 (12.83) | 2821 (9.00) | 219 (14.03) | 3185 (16.58) | 795 ( 9.41) | 238 (12.61) | 924 (7.36) | 0 |  |
| 2022 (%) | 4741 (9.87) | 4920 (15.70) | 354 (22.68) | 3727 (19.40) | 971 (11.50) | 338 (17.90) | 1283 (10.21) | 0 |  |
| 2023 (%) | 3695 (7.70) | 6087 (19.43) | 296 (18.96) | 3898 (20.29) | 1507 (17.84) | 305 (16.15) | 1285 (10.23) | 44 (22.45) |  |
| 2024 (%) | 3706 (7.72) | 8302 (26.50) | 338 (21.65) | 2418 (12.59) | 2293 (27.15) | 282 (14.94) | 1880 (14.97) | 117 (59.69) |  |
| **Occupation distribution of reporters** | | | | | | | | |  |
| Other health- professional (%) | 12015 (25.02) | 2350 (7.50) | 26 (1.67) | 310 (1.61) | 142 (1.68) | 95 (5.03) | 3887 (30.94) | 2 (1.02) |  |
| Pharmacist (%) | 16209 (33.76) | 8570 (27.35) | 393 (25.18) | 2816 (14.66) | 1364 (16.15) | 305 (16.15) | 2660 (21.17) | 31 (15.82) |  |
| Physician (%) | 19793 (41.22) | 20412 (65.15) | 114 (273.16) | 16083 (83.73) | 6939 (82.17) | 1488 (78.81) | 6015 (47.88) | 163 (83.16) |  |
| **Continent distribution** | | | | | | | | |  |
| North America (%) | 19199 (39.98) | 9479 (30.25) | 566 (36.26) | 4042 (21.04) | 2215 (26.23) | 544 (28.81) | 5262 (41.89) | 75 (38.27) |  |
| Europe (%) | 15227 (31.71) | 8605 (27.46) | 661 (42.34) | 5516 (28.72) | 2035 (24.10) | 793 (42.00) | 2628 (20.92) | 35 (17.86) |  |
| Asia (%) | 11496 (23.94) | 12155 (38.79) | 154 (9.87) | 8919 (46.43) | 3945 (46.71) | 422 (22.35) | 4172 (33.21) | 83 (42.35) |  |
| Oceania (%) | 1248 (2.60) | 450 (1.44) | 146 (9.35) | 281 (1.46) | 144 (1.71) | 85 (4.50) | 312 (2.48) | 1 (0.51) |  |
| South America (%) | 725 (1.51) | 601 (1.92) | 34 (2.18) | 366 (1.91) | 94 (1.11) | 43 (2.28) | 163 (1.30) | 2 (1.02) |  |
| Africa (%) | 108 (0.22) | 39 (0.12) | 0 (0.00) | 78 (0.41) | 11 (0.13) | 1 (0.05) | 8 (0.06) | 0 |  |
| Not Specified (%) | 14 (0.03) | 3 (0.01) | 0 | 7 (0.04) | 1 (0.01) | 0 | 17 (0.14) | 0 |  |
| **Distribution of reporting types** | | | | | | | | |  |
| Serious (%) | 43915 (91.46) | 27694 (88.39) | 1383 (88.60) | 18383 (95.70) | 8001 (94.74) | 1668 (88.35) | 11117 (88.50) | 175 (89.29) |  |
| Non-Serious (%) | 4102 (8.54) | 3638 (11.61) | 178 (11.40) | 826 ( 4.30) | 444 ( 5.26) | 220 (11.65) | 1445 (11.50) | 21 (10.71) |  |
| **Outcome distribution** | | | | | | | | |  |
| Life-Threatening(%) | 3801 (7.92) | 1917 (6.12) | 105 (6.73) | 880 (4.58) | 870 (10.30) | 120 (6.36) | 814 (6.48) | 29 (14.80) |  |
| Hospitalization (%) | 21702 (45.20) | 12112 (38.66) | 711 (45.55) | 9261 (48.21) | 3143 (37.22) | 714 (37.82) | 5706 (45.42) | 88 (44.90) |  |
| Disability (%) | 840 (1.75) | 824 (2.63) | 29 (1.86) | 261 (1.36) | 192 (2.27) | 27 (1.43) | 212 (1.69) | 1 (0.51) |  |
| Death (%) | 12530 (26.09) | 6248 (19.94) | 328 (21.01) | 3908 (20.34) | 2234 (26.45) | 507 (26.85) | 2494 (19.85) | 58 (29.59) |  |
| Congenital Anomaly (%) | 20 (0.04) | 10 (0.03) | 0 (0.00) | 1 (0.01) | 5 (0.06) | 0 (0.00) | 5 (0.04) | 0 (0.00) |  |
| Required Intervention (%) | 40 (0.08) | 52 (0.17) | 0 (0.00) | 11 (0.06) | 9 (0.11) | 4 (0.21) | 19 (0.15) | 0 (0.00) |  |
| Other (%) | 38896 (81.00) | 22863 (72.97) | 855 (54.77) | 8224 (42.81) | 4603 (54.51) | 679 (35.96) | 9015 (71.76) | 97 (49.49) |  |
| **Time to onset distribution** | | | | | | | | |  |
| 0-30d (%) | 7807 (16.26) | 5144 (16.42) | 303 (19.41) | 4643 (24.17) | 1419 (16.80) | 403 (21.35) | 2041 (16.25) | 32 (16.33) |  |
| 31-60d (%) | 3564 (7.42) | 1434 (4.58) | 106 (6.79) | 1375 (7.16) | 595 (7.05) | 139 (7.36) | 1088 (8.66) | 8 (4.08) |  |
| 61-90d (%) | 2133 (4.44) | 911 (2.91) | 76 (4.87) | 983 (5.12) | 352 (4.17) | 76 (4.03) | 671 (5.34) | 7 (3.57) |  |
| 91-120d (%) | 1516 (3.16) | 580 (1.85) | 46 (2.95) | 685 (3.57) | 226 (2.68) | 63 (3.34) | 328 (2.61) | 4 (2.04) |  |
| 121-150d (%) | 1056 (2.20) | 398 (1.27) | 37 (2.37) | 520 (2.71) | 137 (1.62) | 46 (2.44) | 175 (1.39) | 5 (2.55) |  |
| 151-180d (%) | 802 (1.67) | 309 (0.99) | 22 (1.41) | 365 (1.90) | 86 (1.02) | 33 (1.75) | 96 (0.76) | 3 (1.53) |  |
| 181-360d (%) | 2237 (4.66) | 925 (2.95) | 83 (5.32) | 1094 (5.70) | 264 (3.13) | 99 (5.24) | 314 (2.50) | 1 (0.51) |  |
| >360d (%) | 1587 (3.31) | 641 (2.05) | 53 (3.40) | 711 (3.70) | 108 (1.28) | 83 (4.40) | 171 (1.36) | 2 (1.02) |  |
| Information loss or abnormality (%) | 27315 (56.89) | 20990 (66.99) | 835 (53.49) | 8833 (45.98) | 5258 (62.26) | 946 (50.11) | 7678 (61.12) | 134 (68.37) |  |
| Median(Q1, Q3) | 50.00  (17.00,134.00) | 31.00  (8.00,104.00) | 43.00  (13.00,130.00) | 41.00  (13.00,126.00) | 39.00  (14.00,94.00) | 43.00  (12.00,139.00) | 41.00  (17.00,82.00) | 28.00  (11.00,88.00) |  |

**
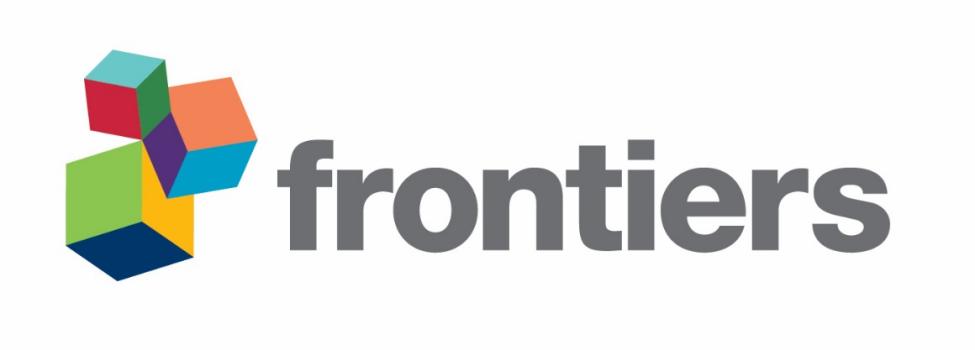
**
